# TRAIL-Based High Throughput Screening Reveals a Link between TRAIL-Mediated Apoptosis and Glutathione Reductase, a Key Component of Oxidative Stress Response

**DOI:** 10.1371/journal.pone.0129566

**Published:** 2015-06-15

**Authors:** Dmitri Rozanov, Anton Cheltsov, Eduard Sergienko, Stefan Vasile, Vladislav Golubkov, Alexander E. Aleshin, Trevor Levin, Elie Traer, Byron Hann, Julia Freimuth, Nikita Alexeev, Max A. Alekseyev, Sergey P Budko, Hans Peter Bächinger, Paul Spellman

**Affiliations:** 1 Department of Molecular and Medical Genetics, Oregon Health and Science University, Portland, Oregon, United States of America; 2 Q-MOL LLC, San Diego, CA, United States of America; 3 The Conrad Prebys Center for Chemical Genomics, Sanford-Burnham Medical Research Institute, Orlando, Florida, United States of America; 4 Inflammatory and Infectious Disease Center, Sanford-Burnham Medical Research Institute, La Jolla, California, United States of America; 5 Department of Biomedical Engineering, Oregon Health & Science University, Portland, Oregon, United States of America; 6 Knight Cancer Institute, Oregon Health and Science University, Portland, Oregon, United States of America; 7 Helen Diller Family Comprehensive Cancer Center, University of California San Francisco, San Francisco, California, United States of America; 8 Computational Biology Institute, George Washington University, Ashburn, Virginia, United States of America; 9 Department of Mathematics and Mechanics, Saint Petersburg State University, Saint Petersburg, Russia; 10 Research Department, Shriners Hospital for Children, Portland, Oregon, United States of America; 11 Department of Biochemistry and Molecular Biology, Oregon Health and Science University, Portland, Oregon, United States of America; Emory University, UNITED STATES

## Abstract

A high throughput screen for compounds that induce TRAIL-mediated apoptosis identified ML100 as an active chemical probe, which potentiated TRAIL activity in prostate carcinoma PPC-1 and melanoma MDA-MB-435 cells. Follow-up *in silico* modeling and profiling in cell-based assays allowed us to identify NSC130362, pharmacophore analog of ML100 that induced 65-95% cytotoxicity in cancer cells and did not affect the viability of human primary hepatocytes. In agreement with the activation of the apoptotic pathway, both ML100 and NSC130362 synergistically with TRAIL induced caspase-3/7 activity in MDA-MB-435 cells. Subsequent affinity chromatography and inhibition studies convincingly demonstrated that glutathione reductase (GSR), a key component of the oxidative stress response, is a target of NSC130362. In accordance with the role of GSR in the TRAIL pathway, *GSR* gene silencing potentiated TRAIL activity in MDA-MB-435 cells but not in human hepatocytes. Inhibition of GSR activity resulted in the induction of oxidative stress, as was evidenced by an increase in intracellular reactive oxygen species (ROS) and peroxidation of mitochondrial membrane after NSC130362 treatment in MDA-MB-435 cells but not in human hepatocytes. The antioxidant reduced glutathione (GSH) fully protected MDA-MB-435 cells from cell lysis induced by NSC130362 and TRAIL, thereby further confirming the interplay between GSR and TRAIL. As a consequence of activation of oxidative stress, combined treatment of different oxidative stress inducers and NSC130362 promoted cell death in a variety of cancer cells but not in hepatocytes in cell-based assays and in *in vivo*, in a mouse tumor xenograft model.

## Introduction

Tumor Necrosis Factor-Related Apoptosis-Inducing Ligand (TRAIL) is considered one of the most effective and reliable inducers of apoptosis in cancer cells [1]. TRAIL, also known as APO-2 ligand and TNFSF10, is a member of the Tumor Necrosis Factor (TNF) family. TRAIL is a type II membrane protein, and, like TNF-α, it can be cleaved from the membrane to produce a soluble, biologically active form [1, 2]. Expression of TRAIL transcripts has been detected in many human tissues, including spleen, lung, and prostate [3]. TRAIL protein is encoded by the *Apo2L* gene located in chromosome 3 (location 3q26). The gene spans 20 kb, contains five exons, and its expression is regulated by interferon (IFN)-α and IFN-β [3].

TRAIL forms homotrimers with a single Zn atom bound by the cysteine residue of each molecule in the trimeric ligand. Zinc stabilizes TRAIL homotrimer formation and is essential for its biological activity [4]. TRAIL induces apoptosis utilizing components of both the extrinsic and the intrinsic apoptotic pathways [1, 2, 5]. In the extrinsic pathway, apoptosis is initiated by interaction of TRAIL with its respective death receptors, DR4 and DR5. These interactions lead to trimerization of the receptor and clustering of the receptor intracellular death domains (DD), followed by the formation of the death-inducing signaling complex (DISC). The DISC formation leads to the recruitment of the adaptor molecule FADD with subsequent binding and activation of the apical caspase-8 and -10. The activated caspase-8 and -10 then cleave and activate the ‘executioner’ caspase-3, -7, and -9. Activation of the ‘executioner’ caspases results in the cleavage of death substrates followed by cell death. TRAIL can also activate the intrinsic pathway by caspase-8-mediated cleavage of the proapoptotic Bid. Truncated Bid then interacts with proapoptotic Bax and Bak that cause the release in the cytosol of mitochondrial cytochrome c and SMAC/DIABLO [1, 2, 5, 6]. The existence of two TRAIL-mediated apoptotic pathways reveals the existence of two different cell types [7, 8]. In type I cells, the apoptotic pathway is independent of the intrinsic pathway and depends on the death receptor-mediated caspase-8 activation followed by the activation of effector caspases. In type II cells, apoptosis is dependent on the amplification of the apoptotic signal *via* the mitochondrial (intrinsic) pathway.

In many cancers, however, the normal apoptotic process is deregulated and the sensitivity to TRAIL is compromised [9–11]. For example, downregulation of TRAIL death receptors DR4 and DR5, overexpression of negative regulators of apoptosis Bcl-2 or Bcl-X(L), and mutations in Bax, Bak, cFLIP, and caspase-8 have been reported to cause TRAIL resistance in various cancer cells [10]. To overcome TRAIL resistance and to identify chemical compounds that can sensitize tumor cells to apoptosis we employed a high throughput screening (HTS) approach followed by *in silico* modeling to expand chemical diversity of TRAIL-sensitizing compounds.

In the present study we demonstrated that one of the discovered compounds, NSC130362, inhibited GSR, a key component of the cellular oxidative stress response. The ability of GSR to influence TRAIL-mediated apoptosis was confirmed by both siRNA and inhibition studies. We also showed that inhibition of GSR by NSC130362 induced oxidative stress in cancer cells but not in human primary hepatocytes as was reflected by a concentration-dependent increase in ROS generation and peroxidation of mitochondrial membrane lipid. Lastly, we demonstrated *in vitro* and *in vivo* that induction of oxidative stress can provide a means for a potent and safe cancer treatment.

## Materials and Methods

### General reagents

All reagents unless otherwise indicated were from Sigma. TRAIL was isolated from *P*. *pastoris* as previously described [12]. GSR activity and GSH detection kits were from Cayman. ATPlite reagent was from PerkinElmer. GSR producing plasmid was a kind gift of Dr. Becker (Justus-Liebig University Giessen). GSR was expressed in BL21(DE3) cells and purified by metal chelating and affinity chromatography on 2’,5’-ADP-Sepharose as described [13].

### Cells

Human prostate carcinoma PPC-1, PC-3, DU145, pancreatic carcinoma SU.86.86, MIA-PaCa-2, PANC-1, BxPC-3, Panc 10.05, Capan-1, lung carcinoma A549, NCI-H1650, mammary epithelial 184A1, MCF10A, and melanoma MDA-MB-435 cells were obtained from ATCC. Breast carcinoma cell lines were obtained from either ATCC or from the laboratories of Drs. Steve Ethier and Adi Gazdar [14]. Bone marrow aspirates or peripheral blood samples were collected from acute myeloid leukemia (AML) patients under the OHSU Institutional Review Board (IRB) approved 4422 research collection protocol which covers *in vitro* drug testing of leukemia cells and genetic studies. Patients signed an IRB-approved written consent form after verbal consent was obtained. Mononuclear cells were isolated from the aspirate using a Ficoll gradient and viability assayed using GuavaNexin reagent. All patients were treated in accordance with the ethical guidelines laid out in the Declaration of Helsinki. AML cells were cultured in RPMI medium supplemented with 10% fetal bovine serum. Human primary hepatocytes were from Lonza. All cells were cultured according to the provider's guidelines.

### High throughput screening (HTS) and virtual screening of small molecule compounds

PPC-1 cells were grown to 90% confluency. The next day, cells were harvested and seeded into a 1536-well plate at a concentration of 250 cells/well using a Biomek FX. After incubation at 37°C for 24 hours, 10 nl of 2 mM compounds were added to compound wells followed by the addition of 10 nl of TRAIL (1 μg/ml) in PBS supplemented with 1 mM MgCl_2_ and CaCl_2_. In addition, 10 nl of 100% DMSO were added to control wells. After overnight incubation at 37°C, 3 μl of ATPLite reagent were added to each well. Plates were then spun down and incubated for 20 minutes at room temperature. The luminescence signal was read by a Perkin Elmer Viewlux plate reader. The screened compounds were from either the NIH Molecular Libraries Small Molecule Repository (MLSMR) (https://mli.nih.gov/mli/compound-repository/mlsmr-compounds/).

#### Small-molecule compounds

MLS0092727 (ML100; PubChem CID 3380841) and its structurally related analogs (PubChem CIDs 781660 and 843346) were purchased as dry powders. Other compounds predicted by virtual ligand screening (VLS) were obtained from the NCI/DTP Open Chemical Repository (http://dtp.nci.nih.gov/repositories.html).

#### Selection of the initial compound’s pharmacophore analogs

The structure of the initial compound ML100 was minimized in 3D using OPLS force field. The optimized 3D structure was then converted into attractive grid-based atom-field potentials. These atom field potentials are generated for individual ligand atoms and account to atom chemical types, atoms coordinates in 3D space, and docking force field parameters used in Q-MOL VLS [15]. The atom-field potentials allow prioritizing hit analogs by adding additional dimensions to the simple chemical similarity metric of a known hit. These dimensions include third coordinate dimension and partial protein-ligand docking dimensions that implicitly link hit molecule to a hypothetical protein target. The search for ML100 pharmacophore analogs was carried out in two steps. First, the Q-MOL chemical fingerprint of the hit was used to search for closest 250 analogs in the NCI library. Then, the atom-field potentials of ML100 were used as docking potentials to dock 250 analogs, which were minimized in OPLS force field, using Monte Carlo simulation in internal coordinates space. The docked analogs were than sorted by their docking energy and first 50 best (minimum energy) analogs were retained and tested in cell based assays.

### Cell viability assays, caspase-3/7 activity analysis, and inhibition studies

Cell viability assay: Cells grown to subconfluency in wells of a 96-well plate were pretreated for 4 h with different concentrations of compounds followed by incubation for 24 h with TRAIL. This sequential treatment efficiently promotes TRAIL-induced apoptosis [16, 17]. The extent of cell lysis (percent of dead cells) was determined by ATP-Lite reagent (Perkin-Elmer).

Caspase-3/7 Assay: The Caspase-Glo 3/7 luminescent assay (Promega) was used to determine caspase-3/7 activity. The resulting luminescence was measured using a plate reader (Promega).

Inhibition studies: Cells were pre-incubated for 4 h with either GSH (10 mM) or general caspase inhibitor Q-VD-OPh (100 μM) (CI) followed by addition of NSC130362 (10 μM) without changing the medium and incubation for 4 h. Next, TRAIL (10 ng/ml) was added to the medium and the cells were incubated for an additional 24 h. The extent of cell lysis was determined as above.

### GSR activity assay and GSH detection analysis

Cells were incubated for 4 h with either NSC130362 (10 or 30 μM) or DMSO in 6-well plate. Next, cells were collected in 200 μl of GR assay buffer (50 mM potassium phosphate buffer, pH 7.5 supplemented with 1 mM EDTA) and disrupted by sonication. Insoluble material was removed by centrifugation. The level of GSR activity and intracellular GSH was determined by GSR activity and GSH detection kit (Cayman), respectively.

### NSC130362 Binding assay

The purified GSR protein at 10 μM concentration was incubated with varying concentrations of NSC130362 in GSR assay buffer (50 mM potassium phosphate buffer, pH 7.5 supplemented with 1 mM EDTA) for 30 minutes at 20°C. 50 μl of each reaction were transferred to a Zebra Spin desalting column (Life Technologies), which was pre-equilibrated with the same buffer, and spun for 2 minutes at 1,500xg. The flow-through was diluted with 0.45 ml of the same buffer and absorbance was measured in a 1-cm path quartz cuvette using the Cary 4000 UV-Vis spectrophotometer (Agilent Technologies). Where needed, NSC130362 was pre-incubated for 2 h at 37°C with GSH in the GSR assay buffer.

### Detection of interaction between NSC130362 and GSH

NSC130362 (300 μM) and GSH (10 mM) were either alone or combined in phenol-free DMEM/10% FBS and incubated at 37°C for 2 h followed by mass spectrometry (MS/MS) analysis. The analysis was conducted on an ABSciex 4000 QTRAP interfaced to a Shimadzu Prominence HPLC. The extent of reaction was determined by the loss of MS/MS spectra for NSC130362 in the presence of GSH. GSH conjugates of NSC130362 were detected with a neutral loss of 129 survey scan and enhanced product ion scans of identified parent ions using LightSight software from ABSciex.

### Small interfering RNA (siRNA) transfection

MDA-MB-435 cells and human hepatocytes were seeded in a 96-well or 6-well plate and allowed to reach subconfluency (50–60%) on the day of transfection. The GSR DsiRNA Duplexes (HSC.RNAI.N000637.12.1 and HSC.RNAI.N000637.12.3) and AllStars Negative Control scrambled siRNA were obtained from Integrated DNA Technologies and Qiagen, respectively. Cells were transfected with 10 nm siRNA in Opti-MEM medium (Invitrogen) using siLentFect lipid reagent (Bio-Rad Laboratories) according to the manufacturer’s protocol. 48 h after transfection, the cells were either treated with TRAIL (10 ng/ml) for an additional 24 h or lysed followed by cell viability measurement or Western blotting analysis, respectively.

### Chromatography/pull down

Epoxy-activated sepharose 6B (GE Healthcare Biosciences) was soaked in distilled water to give approximately 1 ml of final medium. After extensive washing with water, the gel was re-suspended in 1 ml of 0.5 M sodium carbonate, pH 11.0, following the drop-wise addition and stirring of divinyl sulfone (DVS) (0.25 ml); the gel was then incubated with rotation at ambient temperature for 1 h. Next, the gel was washed with water until the flow-through solution had a neutral pH. DVS activates epoxy agarose by introducing reactive vinyl groups into the matrix, which can be used to couple DVS-activated gel with ligands through their hydroxyl groups. NSC130362 and the control compound thymidine, both containing hydroxyl groups, were dissolved in 2 ml of DMSO to 100 mM, mixed with 2 ml of 1 M sodium carbonate, pH 11.0, and covalently linked to 1 ml of DVS-activated gel by incubation with rotation for 24 h at ambient temperature. After extensive washing with 0.5 M sodium carbonate, pH 11.0, containing 10% DMSO, the excess vinyl-active groups were blocked by resuspending the gel in 10 ml of 0.5 M ethanol amine, 10% DMSO, and incubation with rotation overnight at ambient temperature. Finally, the gel was washed with an excess of 20 mM HEPES, pH 7.4, and stored at 4°C.

MDA-MB-435 cells (2×10^8^) grown in DMEM/FBS to subconfluence were dissociated from the plate surface with trypsin, re-suspended in PBS containing 1 mM PMSF, 1 μg/ml each of aprotinin, pepstatin, and leupeptin and disrupted by nitrogen decompression using Parr Cell Disruption Vessel. Insoluble material was removed by centrifugation at 15,000×g for 15 min. The supernatant (about 80 mg of total protein) was split in two equal parts. Each part was chromatographed on either 1 ml NSC130362 or thymidine column. After loading of the supernatant, the columns were rotated at 4°C overnight and consecutively washed with 100 volumes of PBS and 20 volumes of 50 mM ammonium bicarbonate buffer, pH 8.0. The washed agarose was then resuspended in 2 ml of 50 mM ammonium bicarbonate, buffer, pH 8.0, containing 0.1% *Rapi*Gest SF Surfactant (Waters Corporation), 5 mM DTT and rotated for 45 min at 50°C. The thiol groups of the cysteine residues were subsequently blocked by 15 mM iodoacetamide for 30 min in dark conditions, followed by reduction of disulfide bonds with 10 mM DTT for 30 min at ambient temperature. Next, the bound material was digested with 1 μg of trypsin (Pierce) in the presence of 2 mM CaCL_2_ by rotating the sample overnight at 37°C. The digestion solution was then filtered through 0.45 μM filter, adjusted by addition of TFA to 0.5%, and incubated for 45 min at 37°C. After that, the solution was centrifuged at 15,000xg for 10 min and the supernatant was subjected to mass spectrometry (MS) analysis.

### Mass spectrometry (MS) analysis of the digested protein fragments

Digested protein fragments were separated using liquid chromatography with a NanoAcquity UPLC system (Waters), then delivered to an LTQ Velos linear ion trap mass spectrometer (Thermo Fisher Scientific) using electrospray ionization with a Captive Spray Source (Michrom Biosciences). Samples were applied at 15 μl/min to a Symmetry C18 trap cartridge (Waters) for 10 min, then switched onto a 75 μm x 250 mm NanoAcquity BEH 130 C18 column with 1.7 μm particles (Waters) using mobile phases water (A) and acetonitrile (B) containing 0.1% formic acid, 7–30% acetonitrile gradient over 106 min, and 300 nL/min flow rate. Data-dependent collection of MS/MS spectra used the dynamic exclusion feature of the instrument’s control software (repeat count equal to 1, exclusion list size of 500, exclusion duration of 30 sec, and exclusion mass width of -1 to +4) to obtain MS/MS spectra of the ten most abundant parent ions (minimum signal of 5000) following each survey scan from m/z 400–1400.

#### MS data analysis

Version 2012.06 of the Sprot human FASTA protein database (20,237 sequences) was used. We used a sequence-reversed database to estimate error thresholds [18]. The database sequences and their reversed sequences were appended to 179 common contaminant sequences and their reversed forms for a final database of 40,832 sequences. The database processing was performed with SEQUEST/PAWS [19], using no enzyme specificity, average parent mass tolerance of 2.5 Da, monoisotopic fragment ion mass tolerance of 1.0 Da, and variable modification of +16 Da on methionine residues with a maximum of 2 modifications per peptide. Peptide and protein false discovery rates were estimated from decoy protein matches [20].

### Liquid chromatography (LC)-MS/MS Analysis for NSC130362 in mouse plasma samples

Preparation of NSC130362 Samples and Calibrators: Murine plasma samples were thawed at room temperature and 25 μl removed and placed into 1.5 ml microcentrifuge tubes. Calibrators were prepared in PBS at 0, 0.5, 1, 5, 10, 50, 100 and 500 ng/ml, and 25 μl of each spiked PBS calibrator was used. The internal standard, NSC39344 (50 μl of 10 ng/ml in acetonitrile), was added to all tubes. Tubes were vortexed for 5 minutes with a pulsing vortexer, followed by centrifugation at 16,000xg at 4°C for 5 minutes. The clear supernatant was removed from the pellet and transferred to a new 1.5 ml microcentrifuge tube. Samples were dried under vacuum for 20 minutes at 40°C. The residue was re-suspended in 25 μl of acetonitrile:water (1:1), vortexed briefly, and then spun to collect all volume and transferred to autosampler vials with inserts and analyzed by LC-MS/MS. The injection volume was 10 μl. Slopes of standard curves prepared in mouse plasma and in PBS were the same. The lower limit of quantification (LLOQ) was determined to be 0.5 ng/ml having a relative standard deviation of <20%.

NSC130362 and NSC39344 were quantified using an Applied Biosystems QTRAP 4000 hybrid/triple quadrupole linear ion trap mass spectrometer (Foster City, CA) with electrospray ionization (ESI) in positive mode using multiple reactions monitoring (MRM). The mass spectrometer was interfaced to a Shimadzu (Columbia, MD) SIL-20AC XR auto-sampler followed by 2 LC-20AD XR LC pumps. Compounds were infused individually and instrument parameters optimized for each multiple reaction monitoring (MRM) transition. The gradient mobile phase was delivered at a flow rate of 0.4 ml/min and consisted of two solvents, A: 0.1% formic acid in water and B: 0.1% formic acid in acetonitrile. Analytes were separated using a Gemini-NX 3 μ C18 110 Å 50x2 mm column with a 10x2 mm guard column of the same packing and kept at 35°C. The autosampler was maintained at 4°C. Gradient elution was as follows: initial %B of 10% held for 0.1 minutes, followed by an increase to 90% B at 5 minutes, which was held for 2 minutes, dropped back to 10% B over 0.1 minutes, and held at 10% to re-equilibrate for 3 minutes for a total run time of 10 minutes. Data were acquired using Analyst 1.5.1 software and analyzed using Multiquant 2.1.1.

### Animal Research & Ethics Statement

This study was carried out in strict accordance with the recommendations in the Guide for the Care and Use of Laboratory Animals of the National Institutes of Health. The protocol was approved by the Institutional Animal Care and Use Committee(s) (IACUC) of the University of California, Helen Diller Family Comprehensive Cancer Center (Protocol Number: AN092211). Animals were housed in the Helen Diller Family Comprehensive Cancer Center Laboratory Animal Facility with food and water provided *ad libitum* and monitored daily for their well-being. Humane endpoints (e.g. euthanasia on display moribund characteristics such as ruffled coat, little or no activity, eye secretions, and decreased breathing) were in place. All efforts were made to minimize animal suffering during the experiments. At the end of each experiment, animals were euthanized by CO_2_ asphyxiation.

### *In vivo* studies

#### Pharmacokinetic (PK) assay

Oral gavage (p.o.), intra-peritoneal (i.p.), and intra-venous (i.v.) routes of administration were performed on 6–7 wk old female nude mice (Fox N1 nu/nu, Harlan Laboratories). Animals received a single, 10 mg/kg dose in groups of three mice/route of administration. For all three routes of administration, compound NSC130362 was dissolved in DMSO then diluted in saline (0.9% NaCl in water) to 10% DMSO, 90% saline. Blood (80 μl) was collected from the saphenous vein at intervals post-dosing (1 h, 3 h, 8 h, 24 h) in blood collection tubes (Sarstadt CB300) and serum was prepared for analysis. Compounds were detected by time of flight mass spectroscopy.

#### Tumor Xenografts

To evaluate the antitumor activity of NSC130362, we used the heterotopic indirect tumor xenograft model in nude mice (Fox N1 nu/nu, Harlan Laboratories). Early passage MIA PaCa-2 cells were harvested and a cell suspension (1:1 serum free DMEM:Matrigel) was injected subcutaneously (s.c.) into the right flank of anesthetized donor nude mice (10^6^ cells/mouse in 0.1 ml of PBS). When the mean tumor volume was 150 mm^3^, tumor-bearing mice were then be randomized into four treatment groups (5–10 mice per group) and treatment was began with either (I) vehicle alone (DMSO:Saline 1:10, 10 μl/g, p.o. and PBS 5 μl/g, i.p.); (II) ATO dissolved in sterile PBS (10 mg/kg, i.p.); (III) NSC130362 in DMSO:Saline 1:10 (100 mg/kg, p.o.); (IV) NSC130362 (100 mg/kg, p.o.) plus ATO (5 mg/kg, i.p.). Treatment was continued daily for 11 days. Tumor size was determined by external caliper measurement twice weekly. Tumor volume was calculated using the formula: π/6(D1×D1)xD2. At the end of the treatment, animals were sacrificed and the tumors were removed. To analyze the possible toxic effects of treatment, the mouse liver and heart were fixed in 10% neutral buffered saline, embedded in paraffin, and histologically analyzed for signs of toxicity.

### Statistical analysis

The results of the different assays are expressed as mean values based on at least three replicates. The statistical significance of differences in the treatment outcomes was determined by one-way ANOVA or Mann-Whitney U tests.

## Results

### ML100 and its structural analogs potentiated TRAIL-induced apoptosis in cancer cells

**Fig 1:**
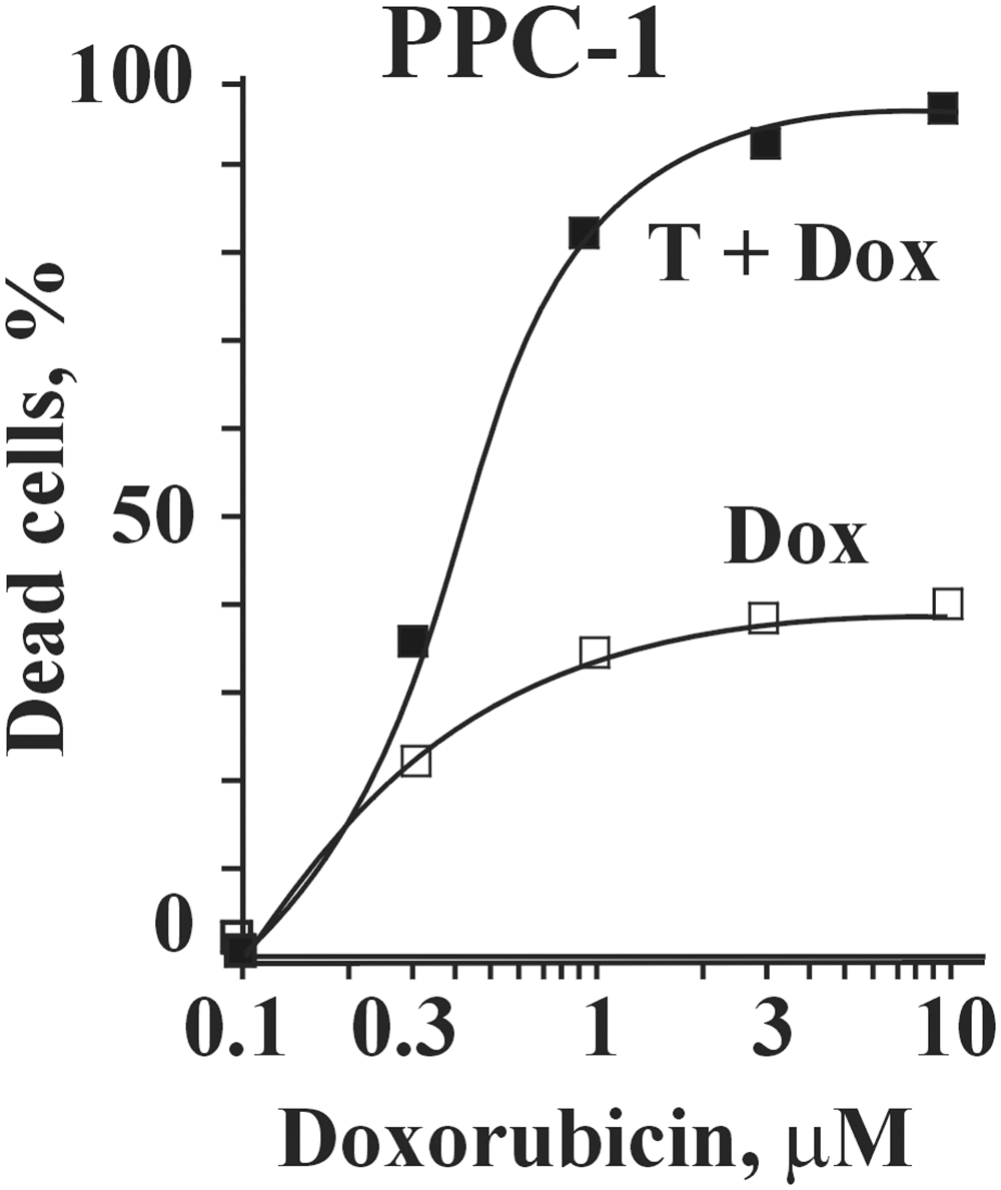
Combined effect of TRAIL and doxorubicin in prostate carcinoma PPC-1 cells. PPC-1 cells grown to subconfluency in 384 well plate were treated with TRAIL (0.1 ng/ml) and increasing concentrations of doxorubicin. The level of cytotoxicity was determined by an ATPLite reagent. T, TRAIL; Dox. doxorubicin. *P* < 0.01.

We sought to identify compounds that potentiate TRAIL activity in TRAIL-resistant cancer cells. For this purpose we developed an HTS assay using human hormone-refractory prostate carcinoma PPC-1 cells. We selected this cell line because the TRAIL-mediated apoptotic pathway in these cells can be readily activated following treatment with cytotoxic agents such as doxorubicin [21] (Fig 1). In addition, PPC-1 cells have functional caspase-3, -8, and -9 as well as other components of the extrinsic (death receptor-mediated) apoptotic pathway, while the intrinsic (mitochondrial) apoptotic pathway is impeded by a mutation in the *TP53* gene [22]. The intact extrinsic apoptotic pathway and the absence of functional p53 increase the probability of identifying chemical compounds that induce apoptosis *via* the TRAIL-mediated extrinsic apoptotic pathway. These features make the PPC-1 cell line optimal for screening chemical compounds that stimulate TRAIL-mediated apoptosis.

More than 200,000 compounds from the NIH MLSMR (https://mli.nih.gov/mli/compound-repository/mlsmr-compounds/) were screened, and 883 compounds that induced cell death in PPC-1 cells in a combined treatment with TRAIL were selected (PubChem AID 1443). A secondary confirmation screen of the identified compounds was carried out in either the presence or the absence of TRAIL to select those compounds that were not cytotoxic in the absence of TRAIL but induced apoptosis in the presence of TRAIL. Only one compound from the selected compound sub-library, ML100, met these criteria (AID 1752).

**Fig 2:**
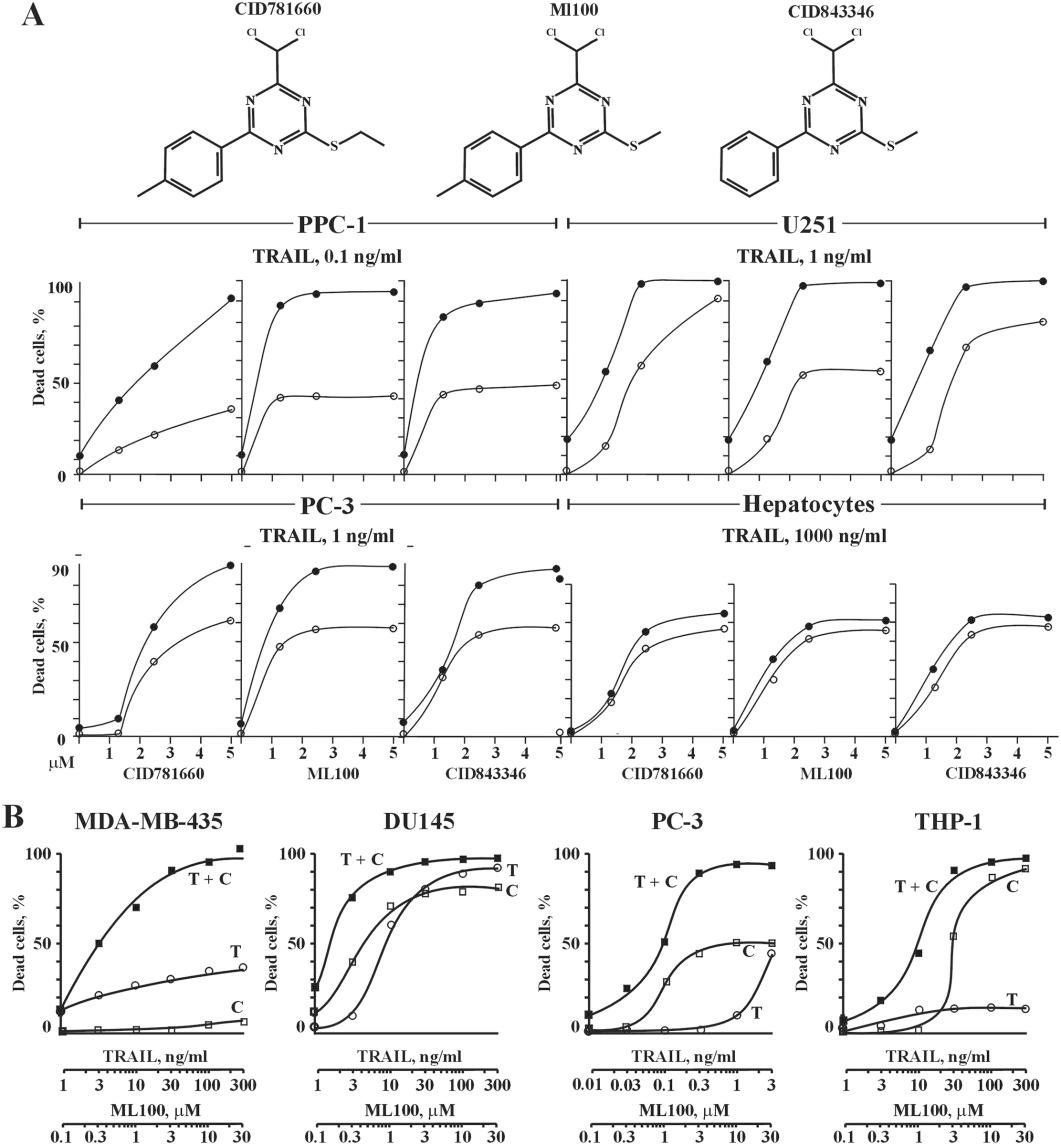
(A) upper panel, the structure of ML100 and its structurally related analogs. Lower panel, effect of ML100 and its structurally related analogs on TRAIL-mediated apoptosis in cancer cells and human hepatocytes. Subconfluent prostate carcinoma PPC-1 and PC3, glioma U251 cells, and human primary hepatocytes in a 96-well plate were pre-incubated for 4 h with the indicated concentrations of compounds followed by TRAIL treatment at the constant concentration of 0.1 ng/ml (PPC-1), 1 ng/ml (PC-3 and U-251), and 1000 ng/ml (hepatocytes) for an additional 24 h. At the end of the treatment, the ratio of dead cells was determined by an ATPLite reagent. Open and closed circles refer to compound sole and compound/TRAIL combined treatment, respectively. (B) isobologram analysis of the combined treatment of TRAIL and ML100 in cancer cells. Subconfluent melanoma MDA-MB-435, prostate carcinoma DU145 and PC-3, and leukemia THP1 cells in a 96-well plate were treated with TRAIL and ML100 at a ratio of 1 ng/ml of TRAIL: 0.1 μM of ML100 (1: 0.1). Cells were pre-incubated with chemicals for 4 h followed by addition of TRAIL and incubation for an additional 24 h. At the end of the treatment, the ratio of dead cells was determined by an ATPLite reagent. T, TRAIL; C, compound.

To confirm the results of the primary and the secondary screening, the dose-dependent activities of ML100 and its structural analogs CID 781660 and CID 843346 (Fig 2A, upper panel) were analyzed in a TRAIL-based cell viability assay employing prostate carcinoma PPC-1 and PC-3, glioma U251 cells, and human primary hepatocytes (Fig 2A, lower panel) (AID 1624, 1746). We selected hepatocytes as normal cells because hepatocytes are one major mechanism of clearance for drugs and are used as a standard to assess toxicity of drugs and other xenobiotics *in vitro* [23]. ML100 and its structural analogs promoted TRAIL activity in all tested cancer cell lines. Although ML100 and its analogs were cytotoxic toward human hepatocytes, the cell viability profiles were similar for both their sole and combined treatments with TRAIL. We conclude that, in contrast to the cancer cells, ML100 and its analogs do not potentiate TRAIL activity in hepatocytes.

To unambiguously assess if the apoptotic response induced by a mixture of TRAIL and ML100 is greater, equal to or smaller than what would have been expected on the basis of the individual activities of the component agents, we performed an isobolographic analysis (Fig 2B) [24]. TRAIL and ML100 synergistically induced apoptosis in MDA-MB-435, DU145, and THP-1 cells at a ratio of 1 ng/ml of TRAIL/0.1 μM of ML100 and in PPC-3 cells at a ratio of 0.01 ng/ml of TRAIL/1 μM of ML100, respectively. For example, in MDA-MB-435 and PC-3 cells, TRAIL and ML100 synergistically induced apoptosis over all of the range of the tested concentrations, while in DU145 and THP-1 cells the synergistic effect of the treatment was seen only at 0.1–0.3 and 0.1–3 μM of ML100, respectively.

### The ML100 pharmacophore analog NSC130362 exhibited potent and safe anti-cancer activity

**Fig 3:**
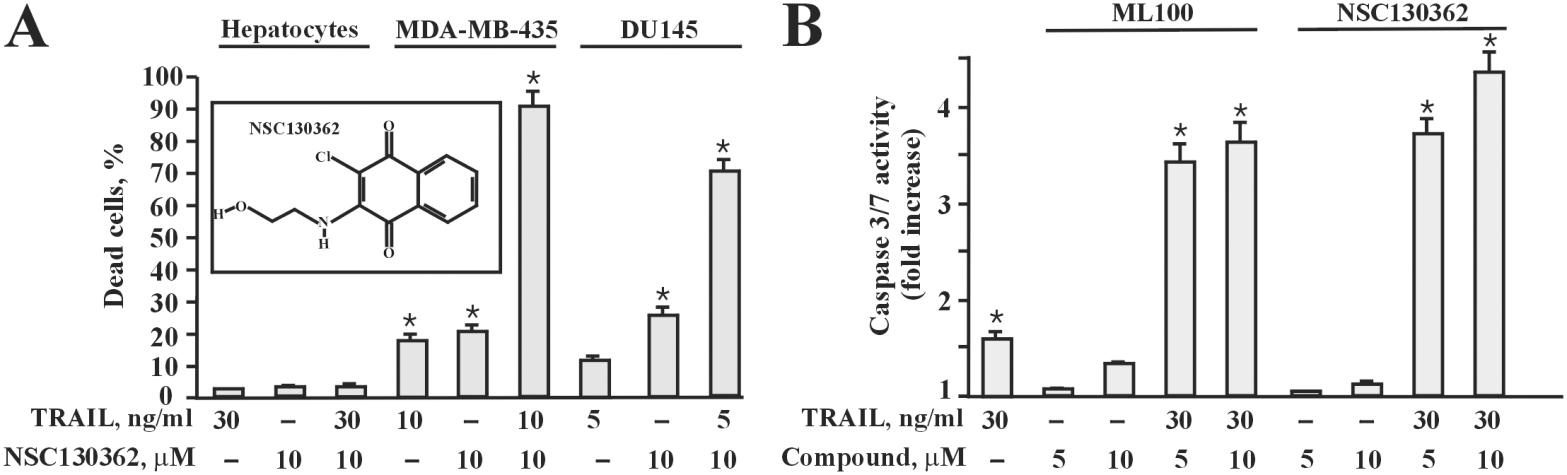
(A) a pharmacophore analog of ML100, NSC130362, exhibited potent anti-cancer activity and was non-toxic to human hepatocytes. The effect of NSC130362 (*inset*) on the viability of MDA-MB-435, DU145 cells and hepatocytes was determined in a TRAIL-based combined treatment as described in the legend for Fig 2. *, *P* < 0.05. (B) both ML100 and NSC130362 synergistically induced caspase 3/7 activity in MDA-MB-435 cells. MDA-MB-435 cells were pre-incubated with ML100 and NSC130362 for 2 h followed by TRAIL treatment for 2 and 6 h, respectively. Caspase 3/7 activity was measured by Caspase 3/7 Lux assay (Promega). *, *P* < 0.05.

To corroborate the role of ML100 in the TRAIL pathway, we employed Q-MOL multidimensional atom-field potentials to predict its pharmacophore analogs from the NCI DTP compound collection. We selected approximately 50 ML100 analogs and tested them in cell-based assays. One of the tested compounds, NSC130362 (Fig 3A, *inset*) induced 75–90% cell death in MDA-MB-435 and DU145 cells but did not affect the viability of human primary hepatocytes (Fig 3A).

To confirm that both ML100 and NSC130362 potentiated apoptotic process in cancer cells we assessed caspase 3/7 activity in treated and untreated cells (Fig 3B). We selected the human MDA-MB-435 melanoma cell line for further analysis because these cells are resistant to TRAIL and our first intention was to find those compounds that sensitize TRAIL-resistant cells to apoptosis, helping to elucidate mechanisms of TRAIL resistance. We observed that the combined treatment of TRAIL with ML100 was more rapid in inducing apoptosis in MDA-MB-435 cells than combination of TRAIL with NSC130362 (data not shown). Based on these data and to maximize the effects of the treatment, we selected different incubation times for ML100 and NSC130362 in the caspase 3/7 activity assay. Our data convincingly demonstrated that the combined treatment of TRAIL with either ML100 or NSC130362, but not their sole treatments, led to a 3.5-4-fold increase in caspase 3/7 activity in MDA-MB-435 cells (Fig 3B).

We also measured caspase-8 activation because caspase-8 is apoptosis-initiating caspase in the TRAIL pathway. However, we could not detect any notable increase in caspase-8 activity after treatment of MDA-MB-435 cells with TRAIL, ML100, NSC130362, or their combinations. This can be explained by the fact that MDA-MB-435 cells are TRAIL resistant and apoptosis in these cells is mediated *via* the intrinsic, caspase-8 dispensable pathway. In addition, activation of caspase-8 is less prominent in type II cells where mitochondria pathway is required to amplify apoptotic signal [7, 8].

### TRAIL-potentiating activity of the identified compounds does not correlate with the level of TRAIL receptors

**Fig 4:**
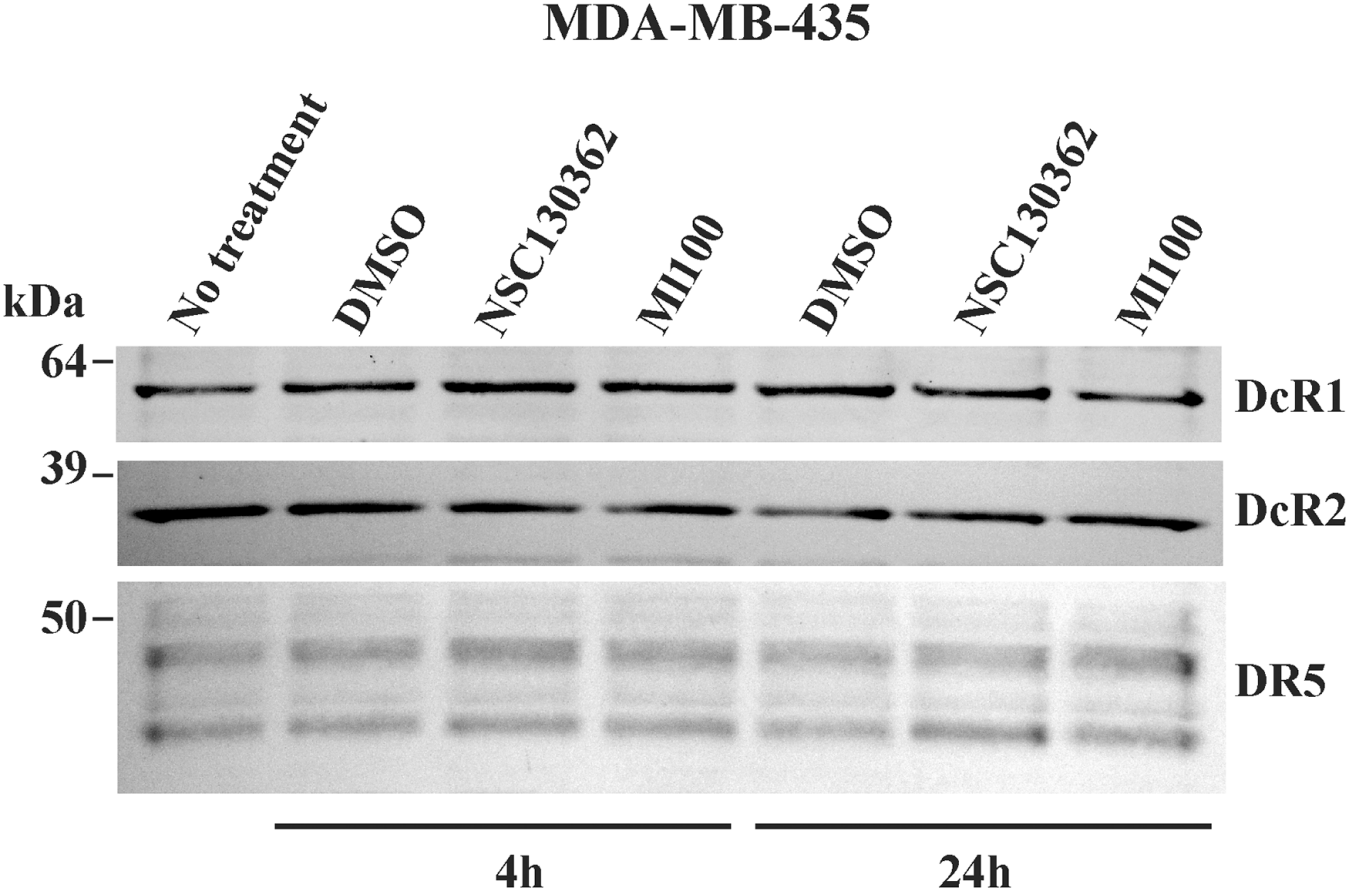
Cell surface expression of DR5 and DcR1/DcR2 in MDA-MB-435 carcinoma cells. MDA-MB-435 cell were pretreated with either ML100 NSC130362 for 4 h and 24 h. Cells were then labeled with biotin and lysed. Labeled cell surface-associated DR5, DcR1, and DcR2 were captured by streptavidin-agarose beads. The precipitated samples were analyzed by Western blotting with the specific respective antibodies and horseradish peroxidase-conjugated secondary antibodies. The level of the cell surface DR4 was below the detection limits. Molecular weight markers are on the left.

We next determined if the level of TRAIL death DR4/DR5 and TRAIL decoy DcR1/DcR2 receptors correlates with the ability of ML100 and NSC130362 to potentiate TRAIL activity in carcinoma cells. For these purposes, MDA-MB-435 cells were pre-incubated for 4 and 24 h with the selected compounds and were labeled with membrane-impermeable biotin. Biotin-labeled cell proteins were captured using streptavidin-agarose beads. The precipitates were analyzed by Western blotting with the DR4, DR5, DcR1, and DcR2 antibodies (Fig 4). Our results showed that neither the level of DR5 nor the levels of DcR1 and DcR2 correlated with TRAIL activity in compound-sensitized cells. We did not detect any DR4 cell surface expression in MDA-MB-435 cells. It is most likely that TRAIL induces apoptosis in MDA-MB-435 cells *via* its binding to DR5. Based on these results we concluded that the tested compounds do not affect the level of both DR5 and DcR1/DcR2. Thus, we conclude that the target protein of both ML100 and NSC130362 is involved in TRAIL-mediated apoptotic signaling downstream of the TRAIL/TRAIL death receptor binding event.

### NSC130362 bound to and inhibited GSR activity

To identify the protein target of NSC130362—the inhibition of which caused the observed cell death in cancer cells—we used pull-down chromatography. First, we coupled NSC130362 and a control compound, thymidine *via* available hydroxyl groups, to an epoxy-activated resin. Thymidine was selected as the control compound because its structure resembles that of NSC130362. Compounds were each immobilized on a solid matrix support in alkaline conditions because these conditions favor covalent bond formation between hydroxyl and epoxy groups. Coupling efficiency was approximately 50% for both compounds as determined by measuring the compound concentration in the flow-through solution after coupling. The compound concentration was estimated by absorbance at 300 nm.

The resulting sorbents were used for isolation of the NSC130362-binding proteins from MDA-MB-435 cells. MDA-MB-435 cells grown to subconfluency were washed and lysed. Equal aliquots of the lysate were chromatographed on each column. After washing of the columns, the bound proteins were digested with trypsin, and digested protein fragments were analyzed by MS (S1 Table). Among the 221 proteins that bound to NSC130362 column and did not bind the thymidine column, GSR was a likely candidate for the protein factor responsible for the observed phenotype.

**Fig 5:**
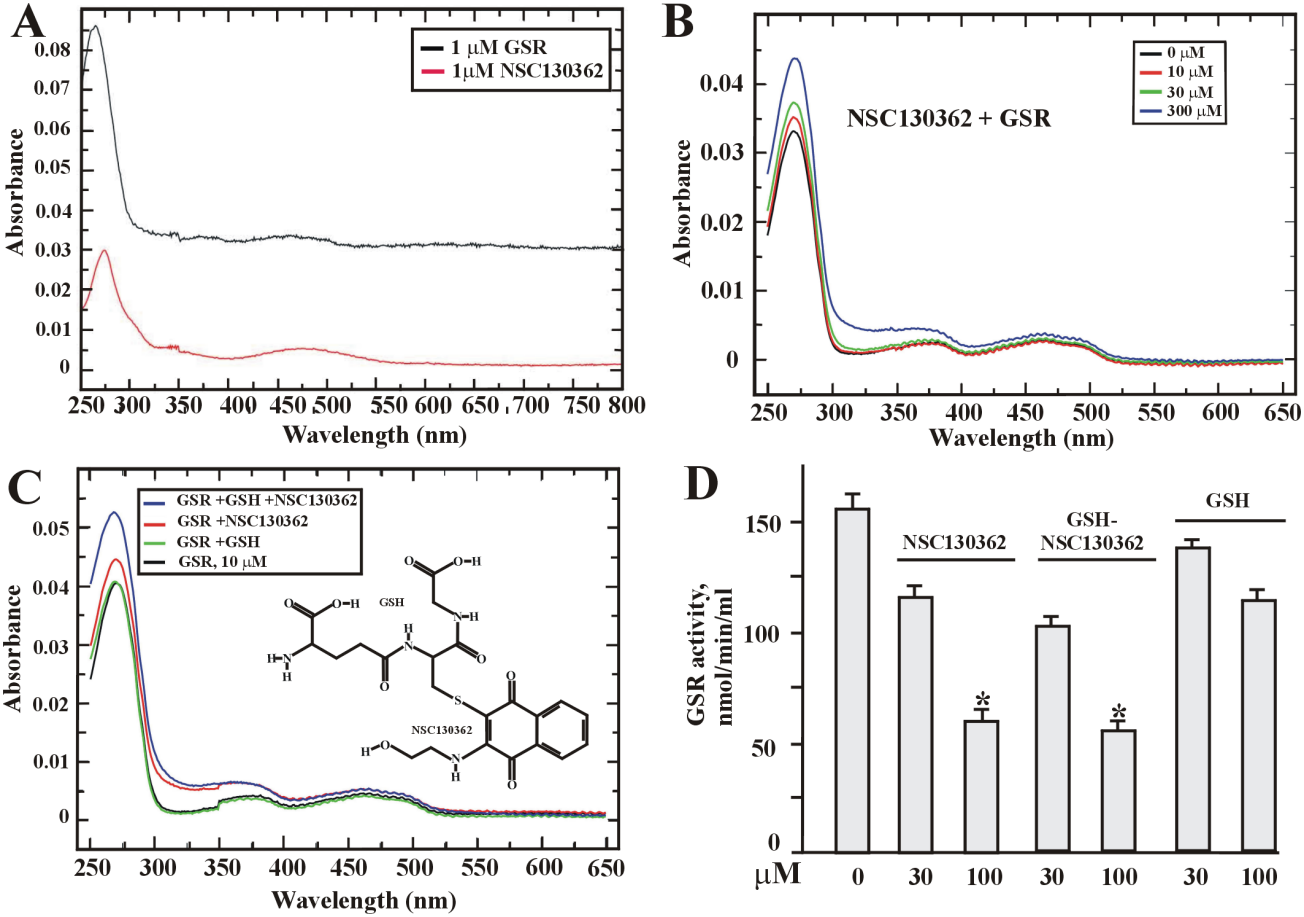
NSC130362 binds GSR in a concentration-dependent manner. (A) GSR and NSC130362 absorbance profile. (B) Absorbance of GSR + NSC130362 in the flow-through after desalting column. (C) *inset*. The structure of the GSH-NSC10362 adduct. Absorbance of GSR + NSC130362 +GSH in the flow-through after desalting column. All absorbance values in (B and C) were corrected for absorbance of individual NSC130362, GSH, and the GSH-NSC130362 adduct in the flow through. (D) GSR activity in the absence or presence of either NSC130362 or the GSH-NSC130362 adduct. *, *P* < 0.05.

To confirm binding between the GSR protein and NSC130362, the direct binding assay was developed (see Materials and Methods). This assay was based on the similar absorbance spectra for the GSR protein and NSC130362 which have peak values around 280 nm (Fig 5A). In this assay, two components were mixed, incubated for 30 min, and desalted to remove the unbound ligand. Thus, if NSC130362 binds to GSR, it will contribute to an increase of absorbance at ~280 nm in the flow through after desalting column. Fig 5B shows that addition of NSC130362 to GSR caused a concentration-dependent increase in absorbance at 280 nm. The data confirmed that NSC130362 indeed binds GSR and its binding affinity is moderate (in the micromolar range). For moderate affinity interactions, this direct binding assay is rather qualitative as it is based on a slow desalting step, during which a significant amount of the ligand can dissociate and be washed away. Thus, these values are not reflecting the amount of a dynamically bound ligand. More sophisticated methods are required to obtain the real value of Kd, as well as of k_ON_ and k_OFF_ constants, and are now in progress.

Next, we investigated if NSC130362 can directly react with GSH. For this purpose, NSC130362 and GSH were incubated for 2 h at 37°C in DMEM/10% FBS followed by MS/MS analysis. According to our analysis, NSC1130362 efficiently reacts with GSH. Two GSH-conjugates were identified by the neutral loss scan. The first and major metabolite was formed by GSH replacing Cl in NSC130362 (Fig 5C, *inset*). The second metabolite was in much lower abundance and there was not enough signal to establish the reaction site. The GSH-NSC13062 adduct was then allowed to bind GSR followed by desalting using spin desalting columns. Surprisingly, we found that this interaction increased the binding of NSC130362 to GSR (Fig 5C), most likely by involving additional interaction sites that exist between GSR and GSH such as electrostatic interactions between GSH carbonyl groups and side chains of Arg^347^ and Arg^37^ in GSR [25].

Lastly, we tested the GSH-NSC130362 adduct’s ability to inhibit GSR activity. Purified GSR (20 μM) was incubated for 4 h at ambient temperature in the presence or absence of NSC130362 (30 and 100 μM) or the GSH-NSC130362 adduct (30 and 100 μM) followed by addition of GSSG and NADPH according to the instructions in the GSR activity kit (Cayman). GSR activity was determined by the level of NADPH oxidation detected spectrophotometrically at 340 nM (Fig 5D). Our data convincingly demonstrate that GSH attachment to NSC130362 does not block its ability to inhibit GSR.

**Fig 6:**
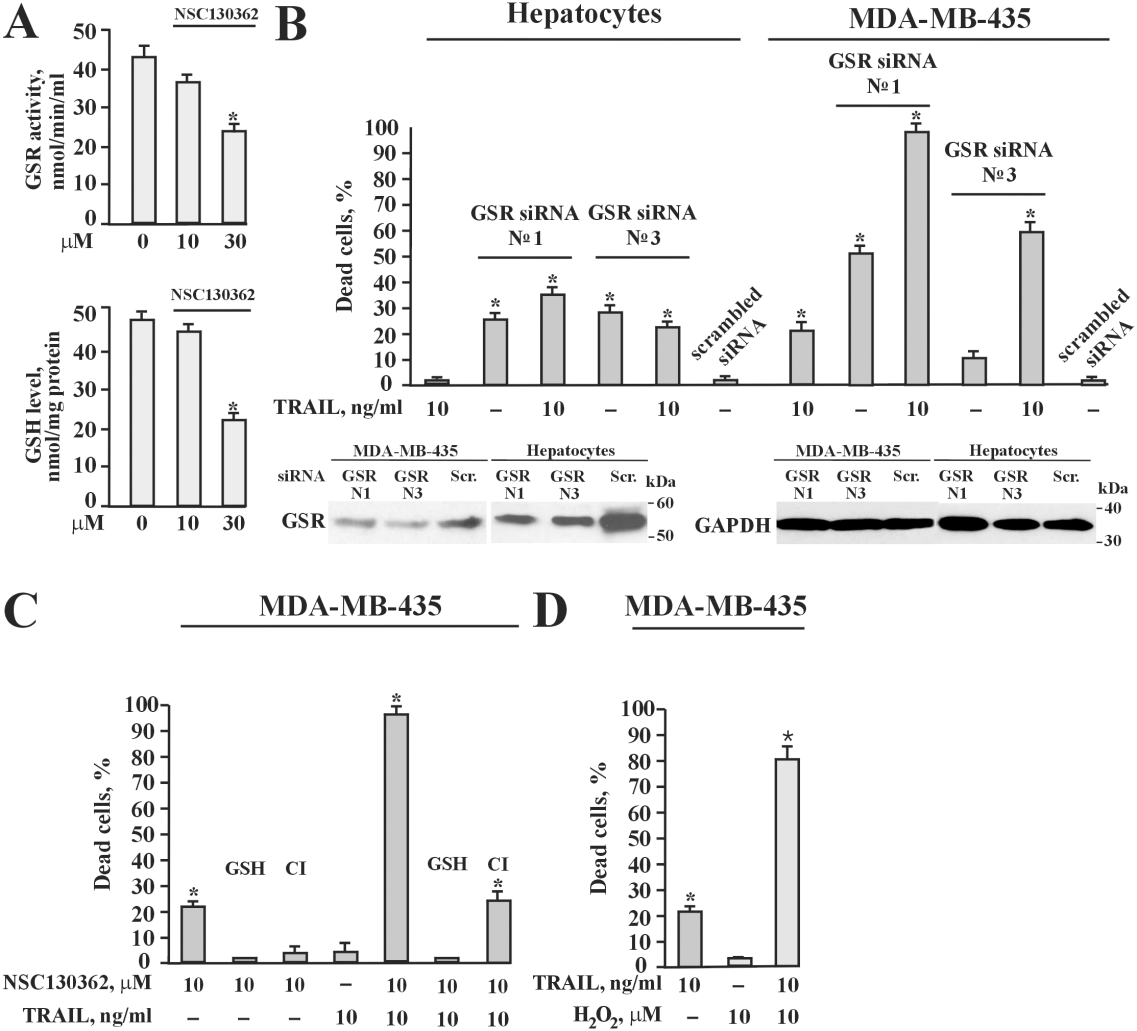
(A) NSC130362 inhibited GSR activity (upper panel) and caused depletion of intracellular GSH (lower panel). MDA-MB-435 cells were treated with 30 μM of NSC130362 for 6 h and the levels of GSH and GSR activity were measured using GSH detection and GSR activity kits (Cayman), respectively. (B) upper panel, GSR siRNA potentiated TRAIL activity in MDA-MB-435 cells but not in human hepatocytes. MDA-MB-435 cells and human hepatocytes grown to subconfluency were transfected with GSR siRNA (10 nM/well) using SilentFect transfection reagent (BioRad). After 2 days, cells were treated with 10 ng/ml of TRAIL for an additional 24 h. Lower panel, Western blotting of GSR. Subconfluent cells were transfected with GSRsiRNA №1 and №3 as well as with scrambled siRNA (10 nM each) in a 6-well plate. Two days after transfection cells were lysed and the level of GSR was analyzed by Western blotting with the GSR antibodies and horseradish peroxidase-conjugated secondary antibodies. (C) GSH but not general caspase inhibitor Q-VD-OPh completely blocked NSC130362 activity. Subconfluent MDA-MB-435 cells in a 96-well plate were pre-incubated for 4 h with either GSH (10 mM) or general caspase inhibitor Q-VD-OPh (100 μM) (CI) followed by treatment with NSC130362 (10 μM) for 4 h and subsequent incubation with TRAIL (10 ng/ml) for an additional 24 h. (D) Hydrogen peroxide potentiated TRAIL activity in MDA-MB-435 cells. Subconfluent MDA-MB-435 cells in a 96-well plate were pre-incubated for 4 h with hydrogen peroxide (10 μM) followed by treatment with TRAIL (10 ng/ml) for an additional 24 h. At the end of all treatments, the ratio of dead cells was determined by an ATPLite reagent. *, *P* < 0.05.

To demonstrate that NSC130362 inhibits the GSR function in cells, the GSR activity and the level of GSH were determined in NSC130362-treated MDA-MB-435 cells (Fig 6A). As a control we used DMSO-treated cells. NSC130362 treatment for 6 h resulted in a concentration-dependent decrease in GSR activity with concomitant drop in the intracellular GSH.

To confirm that GSR is involved in TRAIL-mediated apoptotic signaling in MDA-MB-435 cells, we performed gene silencing and inhibition studies. As shown in Fig 6B, upper panel, silencing *GSR* gene expression potentiated TRAIL activity in cancer cells but not in human primary hepatocytes. Western blotting analysis confirmed a decrease in GSR protein levels after siRNA transfection in both MDA-MB-435 cells and hepatocytes (Fig 6B, lower panel). In addition, NSC130362-induced cytotoxic effects were completely blocked by GSH and only partially inhibited by general caspase inhibitor (Fig 6C). Similar results were obtained with GSH ethyl ester (data not shown). GSH, although to less extent than GSH ethyl ester, can also lead to an increase in the GSH level inside the cell by gamma-glutamyl transpeptidase-mediated GSH homeostasis [26, 27]. Lastly, hydrogen peroxide, a potent oxidative stress inducer, readily potentiated TRAIL activity in MDA-MB-435 cells (Fig 6D). We conclude that GSR is likely a protein target of NSC130362, whose inhibition can induce TRAIL-mediated apoptotic signaling in MDA-MB-435 cells.

### NSC130362-induced ROS and concomitant decreases in GSH levels were responsible for the compound-mediated cell death in cancer cells but not in human hepatocytes

**Fig 7:**
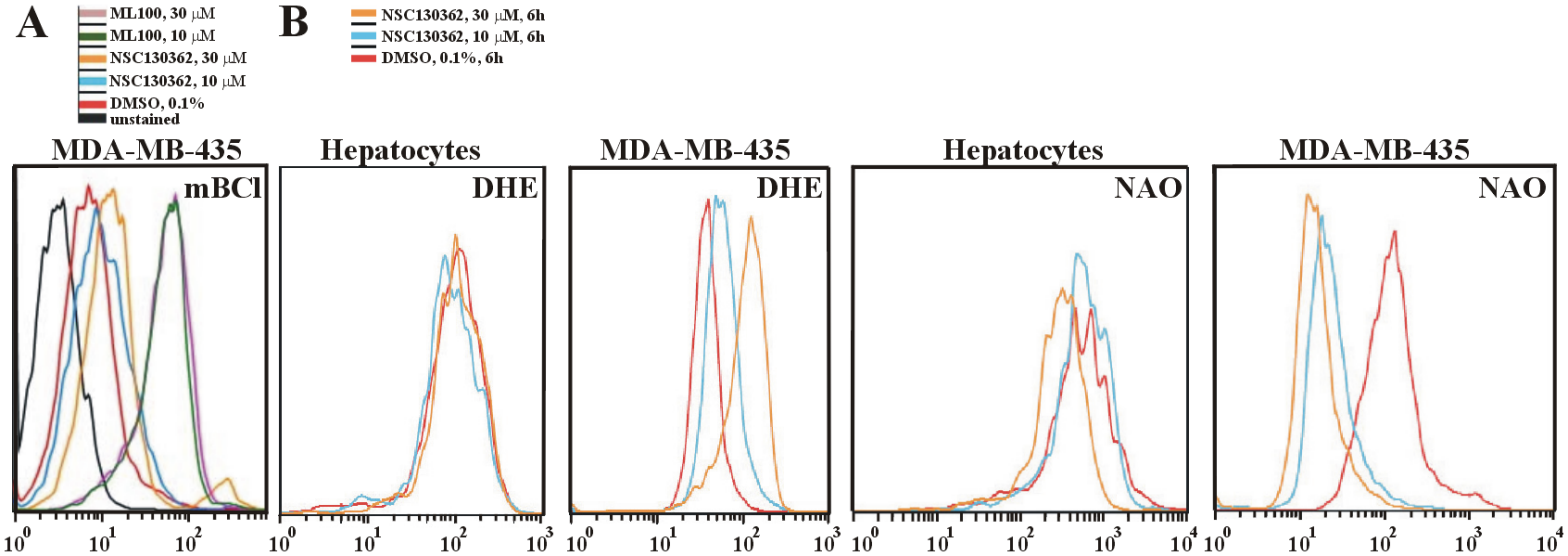
(A) MDA-MB-435 cells that survived after NSC130362 treatment had elevated levels of GSH. Subconfluent MDA-MB-435 cells in a 6-well plate were treated for 6 h with NSC130362 (10 and 30 μM), or DMSO followed by staining with mBCl (40 μM) for 10 min and subjected to subsequent flow cytometry analysis. Mean fluorescence intensity was: 2.55 (unstained cells), 6.47 (DMSO-treated cells), 8.60 (10 μM NSC130362-treated cells), 11.60 (30 μM NSC130362-treated cells), 38.30 (10 μM ML100-treated cells), 37.30 (30 μM ML100-treated cells). (B) NSC130362 induced ROS generation and peroxidation of mitochondrial membrane lipid. Subconfluent MDA-MB-435 cells in a 6-well plate were treated for 6 h with either NSC130362 (10 and 30 μM) or DMSO followed by staining with DHE (10 μM) and NAO (5 nM) for 20 min and subjected to subsequent flow cytometry analysis.

The above-described results suggest that NSC130362 might induce ROS and diminish GSH levels, both of which might be responsible for the compound-mediated cell death. To confirm these assumptions, we treated MDA-MB-435 cells with increasing concentrations of either NSC130362 or ML100 followed by staining live cells with monochlorobimane (mBCl), a cell-permeable dye for quantifying GSH levels in cells (Fig 7A). mBCl is an essentially nonfluorescent dye until it reacts with several low molecular weight thiols, including glutathione. The glutathione conjugate of mBCl can be measured fluorometrically. We specifically selected MDA-MB-435 cells in this analysis because melanoma cells, in general, express high levels of ROS resulting from melanogenesis-related quinonoid metabolism and dysfunctional melanin polymers. These levels of ROS can be easily enhanced to cytotoxic levels if cells are treated with ROS inducers. In agreement with the ability of the tested compounds to induce oxidative stress and subsequent apoptosis, we were able to detect only those cells that had elevated level of GSH, as was evidenced by the increased level of mBCl fluorescence in comparison with that in DMSO-treated MDA-MB-435 cells, and survived after either NSC130362 or ML100 treatment. The similar inverse relationship between oxidative stress induced by quercetin and monobromobimane fluorescence has been shown in earlier studies [28]. Based on these data, we concluded that MDA-MB-435 cells that survived pro-oxidant stress induced by either NSC130362 or ML100 treatment had elevated levels of GSH as compared with MDA-MB-435 cells that did not survive the same treatment. These results confirmed that the level of GSH is one of the factors responsible for the compound-mediated cell death.

To determine if NSC130362 could preferentially induce ROS in cancer cells, we treated MDA-MB-435 cells and hepatocytes with 10 and 30 μM of NSC130362 for 6 h and stained treated cells with dihydroethidium (DHE) and nonyl acridine orange (NAO) (Fig 7B) to detect ROS production and peroxidation of mitochondrial lipid cardiolipin (CL) [29], respectively. Flow cytometry analysis revealed that following treatment with NSC130362, there was a concentration-dependent increase in ROS production in MDA-MB-435 cells but not in human hepatocytes. As expected, increased ROS generation caused cancer-cell-specific CL peroxidation, evidenced by a concentration-dependent left shift of NAO fluorescence.

**Fig 8:**
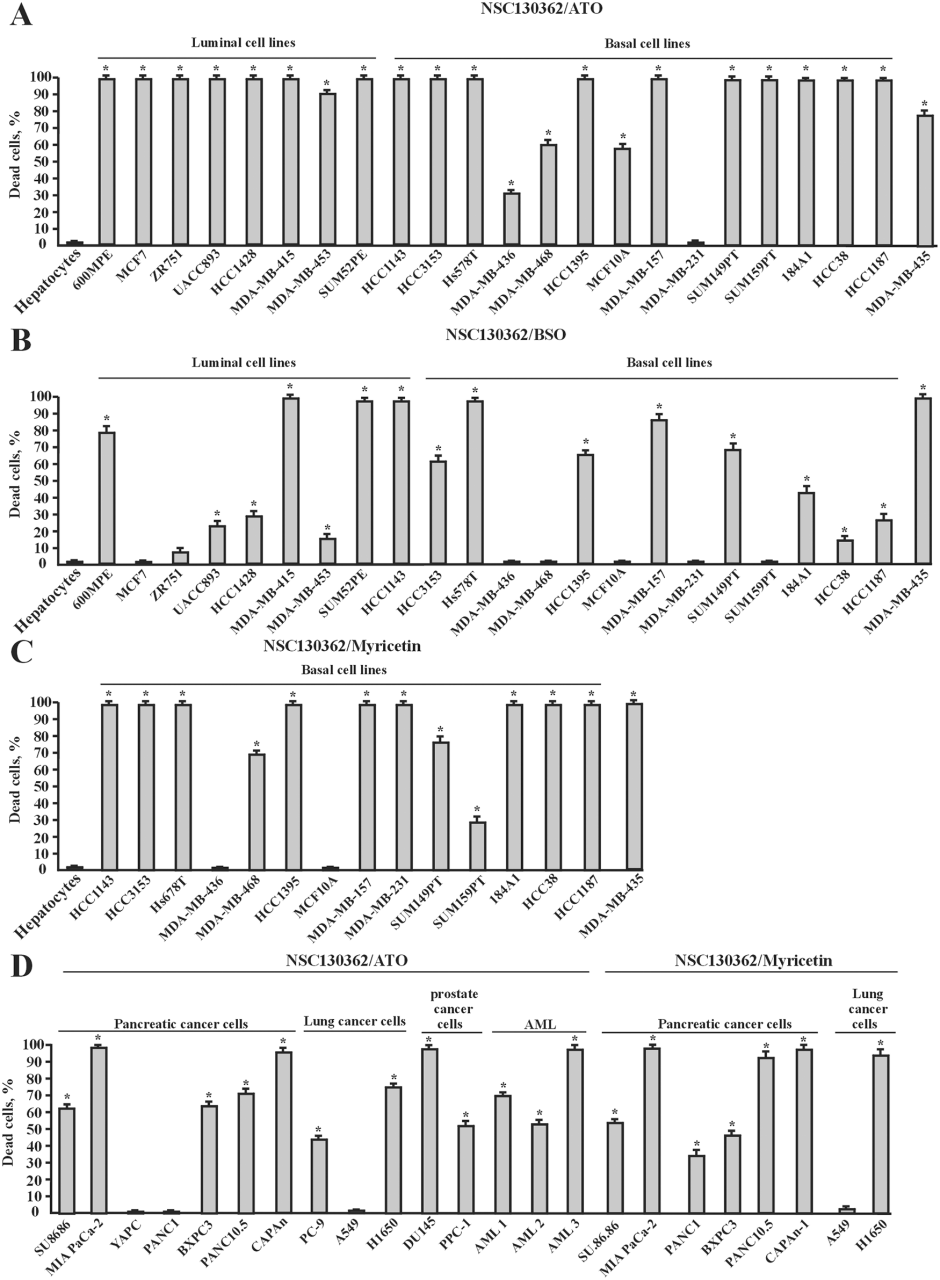
Combined treatment of NSC130362 and oxidative stress inducers ATO, Myr, and BSO efficiently induced apoptosis in a variety of cancer cells but not in primary human hepatocytes. The effect of NSC130362/ATO (A), NSC130362/Myr (B), and NSC130362/BSO (C) combined treatment in breast carcinoma cells and MDA-MB-435 melanoma cells. The effect of NSC130362/ATO and NSC130362/Myr (D) combined treatment in pancreatic, prostate, and lung carcinoma cells as well as in AML cells from cancer patients. Subconfluent cells in a 96-well plate were pre-incubated for 4 h with ATO (3 μM), Myr (100 μM), or BSO (10 μM) followed by treatment with NSC130362 (10 μM) for an additional 24 h. At the end of the treatment, the ratio of dead cells was determined by an ATPLite reagent. *, *P* < 0.05.

Because TRAIL has a poor pharmacokinetic profile in rodents and primates [30], we looked for alternative synergistic treatments with NSC130362. It is well known that increased ROS production might induce oxidative stress, which is linked to mitochondria-mediated apoptosis. Thus, we next determined whether different oxidative stress inducers could potentiate NSC130362 activity in cancer cells and potentially replace TRAIL in combined treatment with NSC130362. As shown in Fig 8A–8D, combined treatment of NSC130362 with different oxidative stress inducers, such as arsenic trioxide (ATO), myricetin (Myr), and buthionine sulfoximine (BSO), caused cell death in a variety of breast, pancreatic, prostate, and lung carcinoma cell lines as well as against human melanoma MDA-MB-435 and AML cells from patients. Importantly, the viability of human primary hepatocytes was not affected by these treatments. Because NSC30362, ATO, Myr, and BSO induce ROS generation and subsequent oxidative stress via different mechanisms, we conclude that the increased level of ROS itself is responsible for the compound-mediated cancer-cell-specific effects on the cell viability.

### NSC130362 exhibited anti-tumor activity *in vivo*

**Fig 9:**
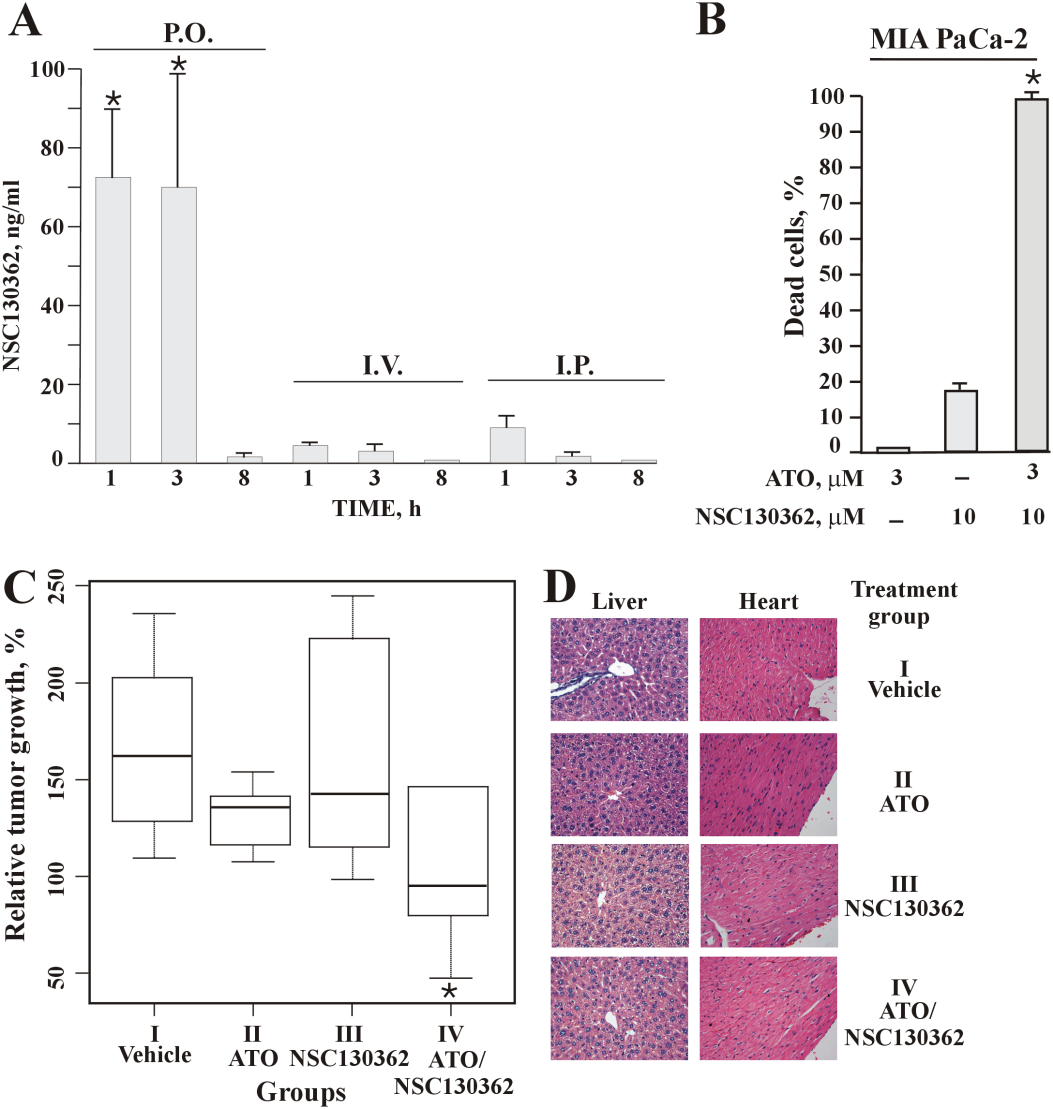
(A) PK studies of NSC130362 in the mouse bloodstream. (B) the synergistic effect of ATO and NSC130362 in MIA PaCa-2 cells. The effect of ATO and NSC130362 on the viability of MIA PaCa-2 cells was determined as described in the legend for Fig 8. (C) the ATO/NSC130362 combined treatment retards growth of MIA PaCa-2 xenografts in immunodeficient mice. (D) H&E-stained sections of mouse liver and heart. Magnification, 40x. *, *P* = 0.03.

The ability of NSC13362 to induce oxidative stress and subsequent apoptosis in cancer cells led us to evaluate its activity in mice. To determine the most effective route of administration we performed pharmacokinetics (PK) studies. NSC130362 was injected intraperitoneally (i.p.), intravenously (i.v.) or delivered by oral administration (p.o.) and blood samples (50 μL) were obtained at the indicated time from the tail vein followed by plasma recovery. The concentration of NSC130362 in the plasma samples was determined by MS analysis (Fig 9A). Our data showed that the most effective route of delivery was by oral administration. In addition, the short half-life of NSC130262 in the mouse bloodstream suggested that the combination of NSC130362 with also short-lived TRAIL (21) could be ineffective in mice despite their clear synergistic anti-tumor effect in cell-based assays. For these reasons, we selected ATO as an agent that could substitute TRAIL and potentiate NSC130362 in *in vivo* studies and confirm the anti-tumor activity and safety to normal cells of NSC130362 in tumor xenograft model.

MIA PaCa-2 cells were selected as target cells in *in vivo* studies because ATO and NSC130362 had a clear synergistic effect in these cells *in vitro* (Fig 9B). These cells were also selected because of their robust engraftment in immunodeficient mice. MIA PaCa-2 cells were xenografted in immunodeficient mice and treatment started when tumor volume was 150 mm^3^. Animals were randomized into four groups. The first (control) group was treated with vehicle solution. The second group received 10 mg/kg i.p. injections of ATO, whereas the third group received p.o. 100 mg/kg of NSC130362. We specifically selected a 10 mg/kg i.p. dosage of ATO because this dosage was the most effective in achieving complete response in earlier studies [31]. The fourth group received combination of ATO (10 mg/kg, i.v.) and NSC130362 (100 mg/kg, p.o.). As expected, tumor growth in the second and third group was similar to that in the control group. Namely, in the control group the relative and relative median tumor volume was 109–235% and 161% of the starting tumor volume, respectively (Fig 9C). In the second and third group, the relative/relative median tumor volume was 107–197% /135% and 98–244%/142%, respectively. On the other hand, in the fourth group the relative/relative median tumor volume was only 47–227%/94% (Fig 9C).

Since the samples were small and the data did not follow a Gaussian distribution, we chose the Mann-Whitney U-test to assess the statistical significance of the obtained *in vivo* data. We chose one-tailed version of the test because we wanted to know if the treatment slowed down the tumor growth. The differences between the second and the control groups (p-value was 0.06) and the third and the control groups (p-value was 0.52) were not significant. In contrast, combination of ATO with NSC130362 in the fourth group caused statistically significant retardation in tumor growth (p-value was 0.017), thus confirming the potency of the ATO/NSC130362 combined treatment *in vivo* (Fig 9C).

At study conclusion tumor, liver and heart tissues from four mice per group were collected, fixed, and H&E stained. Pathological evaluation revealed no evidence of toxicity in normal tissue (Fig 9D).

## Discussion

The goal of this work was to find chemical compounds that selectively sensitize TRAIL-resistant tumor cells to the TRAIL-activated extrinsic apoptotic pathway without affecting normal cells. These compounds would provide useful research tools for interrogating mechanisms of TRAIL-resistance. They also could serve as the basis for future drug development programs to create a new generation of non-toxic anti-cancer drugs that restore sensitivity to endogenous pathways used by the immune system for eradicating tumors.

Some chemotherapeutic agents can augment TRAIL-mediated apoptosis in cancer cells when co-administered with TRAIL *in vitro* and *in vivo* [16, 17, 21, 32–35], but these treatments rely on the intrinsic apoptotic pathway and risk failure because of the acquired defects in the cell death machinery of tumors found in relapsed cancer patients. In addition, these cytotoxic agents can kill normal proliferating cells. Thus, with currently available drugs, cancer chemotherapy affects the survival of both tumor and normal cells, causing patients to suffer from significant toxic side effects.

By employing HTS we identified a promising initial compound, ML100, which efficiently potentiated TRAIL activity in prostate carcinoma PPC-1 cells. Further analysis showed that this compound has the potential to augment TRAIL-mediated apoptosis in TRAIL- and chemotherapy-resistant cancer cells. The screening of chemicals that potentiate TRAIL-mediated apoptosis is not a new approach, but the screening procedure we used was distinct from previous ones in two important ways: (I) we used our potent and safe TRAIL formulation to screen the chemical library [12]; (II) the vast scale of the chemical library (more than 200,000 compounds were tested) was unprecedented. We are convinced that these distinctive features of our screening ensured the identification, for the first time, of a potent initial compound.

ML100 has also been shown to suppress the growth of colon tumor cells lacking either oncogenic beta catenin expression (AID 818) or the tumor suppressor PTEN (AID 827); it also inhibits P450 CYP1A2 (AID 1851) and the assembly of the perinucleolar compartment (PNC) (AID 2417); and it activates the apoptotic arm of the UPR in CHO cells (AID 449763). Some of these activities of ML100 may be relevant to its ability to induce cytotoxicity in cancer cells. For example, mutations that activate beta catenin are commonly found in many forms of malignant human tumors [36, 37] and genetic alterations that reduce PTEN expression have been observed in nearly all types of human malignancies [38, 39]. The P450 cytochromes play key roles in cancer formation, and some P450 forms are selectively expressed in tumors, where they are involved in the activation of various carcinogens [40]. The prevalence of the PNC correlates with malignancies of tumors, more often in cancer cells from solid tissues [41]. Specifically, the PNC is absent in normal breast epithelial cells, while its prevalence reaches 100% in highly metastatic breast carcinoma cells and is associated with poor prognosis [42]. The UPR is also associated with cancer cells, where it is frequently prolonged and leads to the activation of the apoptotic machinery [43]. To summarize, in all of these screens ML100 was identified as an active chemical probe with anti-tumor capabilities, some of which can be related to the same protein target.

Therefore, we hypothesized that a putative ML100 protein target is involved in the TRAIL-mediated apoptotic pathway that is specifically activated in cancer cells. To corroborate our assumption, we used the Q-MOL atom-field potentials tool to identify ML100 pharmacophore analogs in the NCI DTP compound library. Cell-based assays demonstrated that one of the tested compounds, NSC130362, exhibited anti-cancer activity that was both potent and non-toxic to human hepatocytes. This observation is remarkable because most drugs are readily taken up by the liver, where they are subject to metabolic detoxification. As a consequence, chemotherapy has major effect on the viability and function of hepatocytes [23]. The absence of toxic effects of NSC130362 on normal cells has also been shown in *in vivo* studies demonstrating that this compound is not toxic to mice at a concentration as high as 200 mg/kg/injection for 5 days (AID 330). We also demonstrated that either ML100 or NSC130362 and TRAIL synergistically induced caspase-3/7 activity in MDA-MB-435 cells. These data further confirmed preferential action of the identified compounds in the cancer-cell-specific TRAIL-mediated apoptotic pathway. In addition, in contrast to several chemotherapeutic drugs, which potentiate TRAIL activity *via* upregulation of either DR4 or DR5 [16, 17], our results indicate that the relative expression of TRAIL death and TRAIL decoy receptors is not the factor, at least in MDA-MB-435 cells, which restores TRAIL sensitivity in either ML100- or NSC130362-treated cancer cells. Thus, we conclude that the identified compounds are involved in the TRAIL pathway downstream of the engagement of DR4/5.

Identification of this potent and safe anti-cancer compound prompted us to identify its protein target. Chromatography studies showed that GSR could be the factor responsible for the compound-mediated effects. GSR is a key component of the oxidative stress response [44]. The developed NSC130362 binding assay confirmed that NSC130362 indeed binds GSR *via* moderate affinity binding sites. We also showed that NSC130362 directly reacts with GSH and forms the GSH-NSC130362 adduct by Cl-mediated oxidation of GSH sulfhydryl group. Importantly, the formation of the GSH-NSC130362 adduct does not affect the ability of NSC10362 to inhibit GSR, however, it increased the binding of NSC130362 to GSR most likely by introducing additional interaction sites that exist between GSH and GSR [25]. Our cell-based assays also confirmed that NSC130362 inhibited GSR activity in a concentration-dependent manner. The inhibition of the GSR activity most likely caused a drop in the intracellular level of GSH in the compound treated MDA-MB-435 cells. In agreement with the role of GSR in the TRAIL pathway, the *GSR* gene silencing potentiated TRAIL-mediated apoptosis in MDA-MB-435 cells without significant effect on TRAIL activity in human primary hepatocytes. We noticed that GSR siRNA №3 was more cytotoxic in hepatocytes than in MDA-MB-435 and GSR siRNA №1 was more potent than GSR siRNA №3 at inducing cell death in MDA-MB-435, although they were almost equipotent at down-regulating GSR protein levels as was evidenced by Western blotting. These observations can be explained by the influence of several factors on the cell viability. These factors include but are not limited to off-target siRNA effects, cell genotype, epigenetic profile, and state. In addition, Western blotting analysis showed that the effect of siRNA on the *GSR* gene expression is more pronounced in hepatocytes than in MDA-MB-435 cells most likely because hepatocytes have higher level of the GSR protein. All these factors can contribute to the variations in the siRNA-mediated effects on the cell phenotype between different siRNAs and in different cells. The presence of some off-target siRNA effects can also explain why, unlike NSC130362, these siRNAs exhibited some level of hepatotoxicity. However, the shared ability between NSC130362 and two different GSR targeted siRNAs to potentiate TRAIL activity in MDA-MB-435 cells but not in hepatocytes suggests that the target protein of GSR siRNAs and NSC130362 may be identical.

In addition, our chromatography studies showed that there were several other putuaive NSC130362 targets whose inhibition could potentiate TRAIL activity. Indeed, we demonstrated that inhibition of topoisomerase 2 (TOP2A), eukaryotic translation initiation factor 4E nuclear import factor 1 (EIF4ENIF1), CREB Regulated Transcription Coactivator 3 (CRTC3), thioredoxin domain-containing protein 17 (TXD17), and Ras-related C3 botulinum toxin substrate 2 (RAC2) or their interacting proteins promoted TRAIL activity in MDA-MB-435 cells. However, because the effect of NSC130362 on the cell viability in either sole treatment or its combination with TRAIL was fully blocked by GSH, we conclude that the main target, which is responsible for the observed effects, is GSR. In accordance with this conclusion, hydrogen peroxide, a potent oxidative stress inducer, readily potentiated TRAIL activity in MDA-MB-435 cells. Based on these data, we propose the engagement of GSR in the TRAIL apoptotic pathway in, at least some, cancer cells.

The interplay between oxidative stress and TRAIL-mediated signaling has been described in previous studies. For example, the incubation of either bladder cancer cells or melanoma cells with low concentrations of hydrogen peroxide reverses TRAIL resistance [45, 46]. It has also been shown that quercetin, a flavonol that depletes intracellular GSH and induces ROS, can sensitize TRAIL-resistant hepatoma cells to TRAIL-induced apoptosis by multiple mechanisms [47–49]. A more recent paper describes that the altering cellular oxidation/reduction enhances sensitivity to TRAIL by upregulation of DR5 and downregulation of survivin [50]. Moreover, it has been recently discovered that mitochondrial ROS and membrane depolarization mutually regulate one another and are functionally coupled in potentiating TRAIL-induced apoptosis in different tumor cell types [51, 52]. In fact, it has been shown that membrane depolarization, which is associated with intracellular ROS generation and mitochondrial dysfunctions, is an early and prerequisite event in the death receptor-mediated apoptosis. In agreement, many drugs that induce TRAIL activity promote robust depolarization prior to apoptosis [51, 52]. These findings are in accordance with our data showing that NSC130362-induced mitochondrial oxidative stress, as was evidenced by an increase in NAO fluorescence, most likely contributes to TRAIL activity in otherwise TRAIL resistant cancer cells. Because membrane depolarization and ROS production are closely associated with each other, it was expected that TRAIL can also induce mitochondrial superoxide production with subsequent mitochondrial and endoplasmic reticulum stress responses[53]. Collectively, the available data suggest that ROS and membrane depolarization are key factors in TRAIL-mediated apoptotic pathway and any agent, which promotes any of these events, could be a potent TRAIL potentiator.

Despite the activation of the TRAIL pathway in the compound-treated cancer cells, NSC130362-induced apoptosis was also mediated by a caspase-independent process. This was evidenced by the data showing that the NSC19362-induced cytotoxic effect in cancer cells could only be partially diminished in the presence of inhibitory concentrations of the pan-caspase inhibitor Q-VD-OPh. Caspase-independent apoptosis induced by ROS has also been reported in previous studies [54]. Death receptor triggering can also induce cell death by caspase-independent, necrotic pathway [55]. The detailed underlying mechanism of relationships between NSC130362-mediated ROS and the TRAIL pathway is currently under investigation.

Because TRAIL is rapidly eliminated from the bloodstream of rodents and nonhuman primates [30], we also looked for a viable therapeutic alternative to TRAIL in cancer treatment. Elevated levels of ROS and subsequent oxidative stress are hallmarks of carcinogenesis and metastasis [56]. Recent studies convincingly demonstrated that elevated levels of ROS can be exploited *in vitro* and *in vivo* to specifically target cancer cells while sparing normal cells [57, 58]. Because our data showed that NSC130362 treatment caused both dose-dependent accumulation of ROS and peroxidation of the mitochondrial lipid CL in MDA-MB-435 cells but not in human hepatocytes, we propose that NSC130362 could specifically induce cell death in cancer cells. To test if combining NSC130362 with different oxidative stress inducers could be a potent and cancer cell-specific treatment, we analyzed various breast, pancreatic, prostate, and lung carcinoma cell lines, along with melanoma MDA-MB-435 and AML cells from patients. To confirm tumor selectivity, we also treated human primary hepatocytes. To induce oxidative stress, we employed three oxidative stress inducers, ATO, BSO, and Myr. Our results convincingly demonstrated that induction of oxidative stress selectively potentiated the cytotoxic activity of NSC130362 in cancer cells without any notable effect on the viability of human primary hepatocytes. Mammalian cells have two electron donor systems, the thioredoxin (Trx) system and the GSH system that regulate cell metabolism, motility, viability, and reproduction [56]. The stress inducer ATO and the naturally occurring flavonol Myr irreversibly inhibit Trx reductase, thereby inactivating the Trx system [59, 60], while the stress inducer BSO is an inhibitor of GSH synthesis through the irreversible inactivation of gamma-glutamylcysteine synthetase [61]. Based on our cytotoxicity data, we posit that the strategy to inhibit both cellular redox systems is an efficient approach to selectively target cancer cells.

The potent and safe to primary hepatocytes combined treatments of NSC130362 and oxidative stress inducers against a variety of cancer cells as well as the effect of NSC130362 on the TRAIL pathway undoubtedly warranted additional studies using mouse models. The short half-life of NSC130362 in the mouse bloodstream greatly diminished the possibility of obtaining any noticeable effect of its combination with also short-lived TRAIL [30] on tumor growth in mice. For these reasons we decided not to test the combination efficacy of TRAIL and NSC130362 in animal xenograft tumor model as it was shown, for example, for wogonin and TRAIL [62]. Recently, we identified several NSC130362 analogs that have anti-tumor activity and safety to hepatocytes comparable to those of NSC130362. Their pharmacokinetic profile is currently under investigation. As soon as we identify stable NSC130362 analogs, we will test them in combination with TRAIL *in vivo*. In the current studies, we selected ATO as an agent that could substitute TRAIL in *in vivo* studies and confirm the anti-tumor activity and safety to normal cells of NSC130362 in mice. In agreement with the cell-based assays, combination of ATO and NSC130362 retarded growth of MIA PaCa-2 xenografts. Importantly, this combined treatment was not toxic to normal tissue as was evidenced by the H&E staining of liver and heart tissue sections, which are the most sensitive to oxidative stress [23, 63].

In summary, we have demonstrated that phenotypic TRAIL-based HTS and *in silico* methods can be employed to identify chemical compounds that specifically induce cytotoxicity in cancer cells while sparing normal cells. In our study we identified a specific inhibitor of GSR activity, which, when combined with other oxidative stress inducers, may provide the basis for a potent and non-toxic cancer therapy. Our results suggest that increased ROS generation in transformed cells, compared with normal cells [56], is the primary cause for the selectivity and the potency of the described treatment.

## Supporting Information

S1 Table NSC130362 bound proteins. (XLS)
